# Foreign Correspondence

**Published:** 1868-07-15

**Authors:** J. W. Freer


### *FOREIGN CORRESPONDENCE.*

---

BY PROF. J. W. FREER.

DUBLIN, June 7th, 1868.

J. ADAMS ALLEN, M.D.—*Dear Doctor* :—I arrived in the city of Dublin on the 25th ult. We had an unusually short passage — only nine days from New York city to Queenstown — for which favor I am duly thankful, as with me the sea does not improve on acquaintance. In my journeyings I have the pleasure of being associated with Dr. Charles G. Smith, to whose observing and inquiring disposition I am indebted for more enthusiasm in sight seeing than I would be able to get up on my own account. Our first professional visit was to the Medical Department of venerable old Trinity By means of letters of introduction presented us by our esteemed friend, Dr. Dyas, of Chicago, we were at once placed "*en rapport*" with the Faculty, for whose kindness and attentions we are under many obligations. Our time being limited, we confined ourselves mainly to the examination of the museum, which, through the politeness of Dr. Conner, the curator, we were enabled to look over quite thoroughly and understandingly. The collection is very large, and embraces almost every thing which can possibly serve to illustrate natural science. Prof. R. W. Smith's collection of bones is particularly unique and instructive. We were shown three specimens showing union by bone of fractures within the capsule of the hip joint; but in each instance there had been impaction. Also an instance of reunion by bone of a fractured patella. A remarkable specimen of stalactiform exostosis was shown of an individual who died at the age of one hundred years, every joint of whose skeleton is anchylosed, excepting the right wrist; this is said to have escaped the accident because of his inveterate habit of whist playing, which ruling passion remained strong to the last. The joints are literally bridged over by adventitious bone having taken the place of the connective tissues of some of the muscles, as the complexus of the neck is now represented by bone. In fact, he must at some time have been confronted by the head of Medusa. Another remarkable piece in this collection is the skeleton of a man of the height of eight feet six inches, *without stockings*. He died at the age of twenty-three years. The bones are evidently in a pathological condition, for the shafts of the long bones are so soft as to be readily penetrated by the blade of a pocket knife. Altogether this is a very rare collection.

We next presented ourselves at the doors of the College of Surgeons, where we were kindly received by Prof. Hargrave, a venerable gentleman of prepossessing appearance and kindly manners. He showed us around a few moments, and then introduced us to Prof. Barker, who selected and explained to us the representative and rarest specimens of this classified and truly magnificent assemblage of exponents of anatomy, physiology, and pathology. Specimens for practical use in teaching are more numerous and better in this than in the museum of Trinity. On the next day we were honored with an invitation to visit Stephens Hospital, where we were fortunate enough to meet with a full corps of medical and surgical gentlemen, called together on the occasion of its being operating day. Prof. Robert McDonald officiated. The principal operation was the removal of a schirrous breast. He is a graceful, and no doubt a skillful operator — however, the removal of a breast is scarcely a test of the latter qualification. We were so fortunate, on this occasion, as to make the acquaintance of Prof. McDonald, for whose subsequent kindness and attention we can not express too much obligation. He showed us the most interesting cases in his wards, among which was a case of epilepsy of eight years' standing, now under complete control by the use of *Bromide of Potassium*. He is a firm believer in the efficacy of this drug in this horrible affection, but uses it in large doses — say a drachm every twenty-four hours. I was happy to learn that the Dr. is an uncompromising opponent of the indiscriminate use of *Mercury* in syphilis. He discoursed very learnedly and interestingly on this subject, the principal points of which Dr. Smith has taken notes of, and will communicate in due time.

Stephens Hospital has 300 beds, and notwithstanding it was built long ago, is well ventilated and salubrious. "The Misericordia" is a new hospital, and built after modern notions of ventilation, and, in fact, is a model of beauty and perfection, We were not fortunate enough to meet the attending staff, but were shown about by the *interne.*

I had an opportunity of exhibiting blood corpuscles with my illuminator, and am happy to say that it performed well, much to the astonishment of the beholders. Prof. McDonald invited us to his house for that purpose, where every facility was offered for a successful demonstration. I also showed it to Prof. John Hughes Bennett, of Edinburgh, who seemed very much surprised at the result, and made a drawing of human blood corpuscles with the newly discovered elevation in the centre of the concavity of the discs.

Altogether, we were very much pleased with Dublin and Dublin people. The city contains nearly 460,000 inhabitants, and is, for the most part, regularly laid out. The river Liffy passes through the centre, and is frequently spanned by beautiful arched stone bridges. The inhabitants seem more thrifty here than elsewhere in Ireland; there is less of obtrusive poverty than at Queenstown, Cork, and the interior. In fact, in some parts, the beggars follow me like hungry wolves, and will not be appeased without some pennies. To the reflecting mind, the contrast in the condition of the different classes in Ireland is painful to behold. Some people seem to have all they desire, and more than they need; but this otherwise fine picture of competence and contentment is spoiled by the ever present skeleton-limbed aboriginal heirs of the soil.

